# Advances in Biologically Applicable Graphene-Based 2D Nanomaterials

**DOI:** 10.3390/ijms23116253

**Published:** 2022-06-02

**Authors:** Josef Jampilek, Katarina Kralova

**Affiliations:** 1 Department of Analytical Chemistry, Faculty of Natural Sciences, Comenius University, Ilkovicova 6, 842 15 Bratislava, Slovakia; 2 Department of Chemical Biology, Faculty of Science, Palacky University Olomouc, Slechtitelu 27, 783 71 Olomouc, Czech Republic; 3 Institute of Chemistry, Faculty of Natural Sciences, Comenius University, Ilkovicova 6, 842 15 Bratislava, Slovakia; kata.kralova@gmail.com

**Keywords:** graphene, graphene quantum dots, graphene oxide, graphene oxide quantum dots, reduced graphene oxide, nanocarriers, drugs, agrochemicals

## Abstract

Climate change and increasing contamination of the environment, due to anthropogenic activities, are accompanied with a growing negative impact on human life. Nowadays, humanity is threatened by the increasing incidence of difficult-to-treat cancer and various infectious diseases caused by resistant pathogens, but, on the other hand, ensuring sufficient safe food for balanced human nutrition is threatened by a growing infestation of agriculturally important plants, by various pathogens or by the deteriorating condition of agricultural land. One way to deal with all these undesirable facts is to try to develop technologies and sophisticated materials that could help overcome these negative effects/gloomy prospects. One possibility is to try to use nanotechnology and, within this broad field, to focus also on the study of two-dimensional carbon-based nanomaterials, which have excellent prospects to be used in various economic sectors. In this brief up-to-date overview, attention is paid to recent applications of graphene-based nanomaterials, i.e., graphene, graphene quantum dots, graphene oxide, graphene oxide quantum dots, and reduced graphene oxide. These materials and their various modifications and combinations with other compounds are discussed, regarding their biomedical and agro-ecological applications, i.e., as materials investigated for their antineoplastic and anti-invasive effects, for their effects against various plant pathogens, and as carriers of bioactive agents (drugs, pesticides, fertilizers) as well as materials suitable to be used in theranostics. The negative effects of graphene-based nanomaterials on living organisms, including their mode of action, are analyzed as well.

## 1. Introduction

One way to distinguish and classify nanomaterials (NMs) is according to their dimension: zero (0D)-, one (1D)-, two (2D)- or three (3D)-dimensional crystal structure [1,2]. The same materials differ, significantly, in their properties, depending on their dimensional configuration. Nowadays, 2D-NMs are becoming very popular, in terms of their applications.

**Figure 1:**
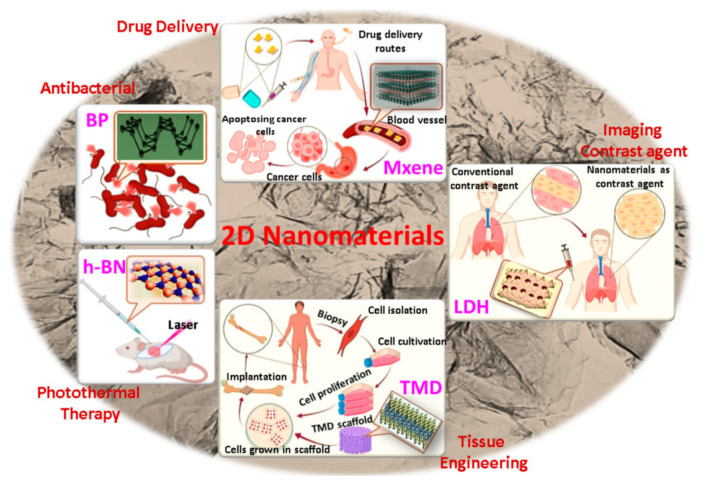
Potential biomedical applications of two-dimensional graphene-based nanomaterials. Adapted from [17], copyright 2022, MDPI.

Such 2D-NMs have a leaf structure, with strong in-plane bonds and weak van der Waals between layers, large surface area, and anisotropic physicochemical properties [3]. They have transverse dimensions >100 nm, and their thickness is usually <5 nm [4]. The history of 2D-NMs begins in 2004, when Novoselov et al. [5] prepared graphene (GR) from graphite. GR is a single-carbon crystalline-carbon film, with various unexpected/unique properties (high surface area [6,7], excellent electrical conductivity [8], strong mechanical strength [9,10], and thermal conductivity [11]), plus, especially, the fact that it can be easily functionalized and modified [12,13,14,15,16,17]. Further research has led to the discovery of a large number of other 2D-NMs [18], such as graphitic-carbon nitride [19,20,21], silicate clays [22,23], layered double hydroxides [24,25,26], transition-metal dichalcogenides [27,28], etc. These 2D-NMs have a wide range of applications, from chemical productions [29,30], optoelectronics [31], sensors [32,33], and energy [34,35], to biomedicine [36,37,38,39], where they have become extensively studied as biologically active substance delivery systems, biosensors, or multimodal imaging materials, see Figure 1. It is important to mention that graphene-based nanomaterials (GBNs) have their own antimicrobial and anticancer activity and are also used for tissue engineering [36,37,40,41,42,43,44,45,46,47]. Their 2D nanostructure gives these NMs special physicochemical properties and biological behaviors, such as cell entry through endocytosis, as well as specific biodistribution, biodegradation, and excretion, which lead to their use in various biomedical applications [17,48,49,50,51,52,53,54,55,56] as well as for the treatment of plants [57,58,59,60].

**Figure 2:**
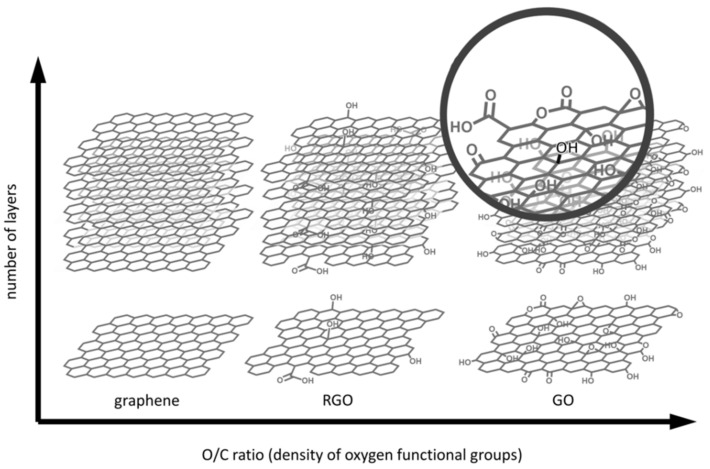
Structural overview of graphene-based materials. Adapted from [62], copyright 2021, MDPI.

The structure (see Figure 2) of GBNs determines their chemical and physical properties. GBNs actually consist of an aromatic carbon lattice (in one or more layers) and, depending on the degree of oxidation (i.e., materials, such as graphene oxide (GO) and reduced graphene oxide (rGO)), a different number and type of oxygen functional groups. However, all GBNs have an extremely large surface area, which, together with the specific structure and degree of oxidation, provide GBNs the capacity and flexibility of loading many types of compounds that are bound by non-covalent interactions (π-π stacking, hydrophobic interaction, and hydrogen bonding) [56,61].

**Figure 3:**
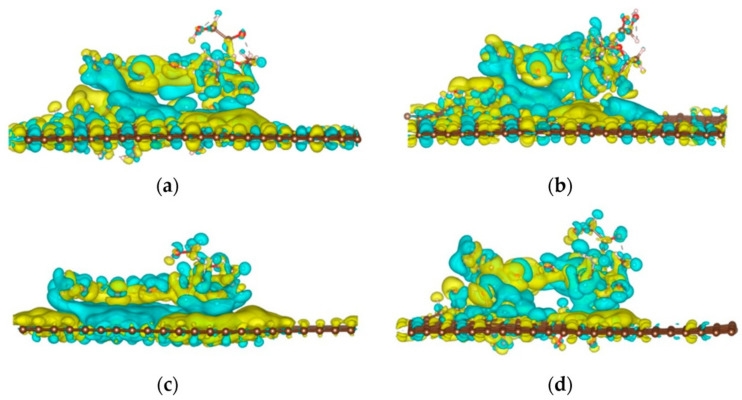
Differential charge density of different molecular graphene surfaces. (**a**) G-DOX; (**b**) GO-OH-DOX; (**c**) GO-O-DOX; (**d**) GO-OH-O-DOX. The yellow and blue areas represent an increase and decrease in charge density, respectively. Adapted from [63], copyright 2022, MDPI.

One of the most used cytostatics is doxorubicin (DOX), with an anthraquinone structure. Song et al. studied the interactions of DOX with the graphene system. It was observed that, when DOX was adsorbed onto pristine GR, it transferred 0.04 electrons to DOX. When DOX was adsorbed onto GO, 0.05 electrons were transferred from DOX to GO-O and the GO-OH-O surface, respectively. Additionally, 0.07 electrons are transferred from DOX to the GO-OH surface [63], see Figure 3. Thus, using density functional theory calculations, it was confirmed that different *O*-moieties had different affinities for DOX. The order of the different GBN systems was as follows (in terms of adsorption energy): G-DOX > GO-OH-DOX > GO-OH-O-DOX > GO-O-DOX. Therefore, increasing the ratio of hydroxyl to epoxy groups can increase the DOX loading capacity of GO. A broad study on the interactions of 2D-NMs, especially GBNs, with biomolecules was published by Chen et al. [64]. In-depth in vivo, in vitro, and in silico analysis revealed that hydrophobic GBNs strongly interact with hydrophobic protein residues, phospholipid membranes, and nucleic acids. Adsorption of proteins on GBNs can cause structural changes and localized unfolding. GBNs can penetrate through cell membranes and extract phospholipids, resulting in membrane disruption. Nucleic acids form stabilizing π–π interactions, different for various 2D-NMs. This knowledge creates space, for example, for the creation of specific nanocarriers, for various ssRNAs in vaccine mRNA. It is important that GOs have the ability to release drugs in an acidic environment (tumors and inflammatory tissues), therefore, they have the potential for chemically mediated targeted therapeutic release [64].

The unique structure of GBNs also causes these NMs to show a significant photothermal effect. In addition to energy and industrial applications [23,24,25,26,27,28], the benefits/applications of GBNs in photodynamic (PDT), photothermal therapy (PTT), and photothermal antibacterial protection are expected [56,65,66,67]. These specific optical properties of GBNs, especially absorption of NIR radiation [68,69,70], may allow another mode of targeted delivery, via photothermal drug release, and also allow a new targeted therapeutic modality, via photothermal ablation [64].

The number and type of *O*-moieties in GBNs affect not only the loading capacity and the spectrum of loaded molecules but also the toxicity of GBNs, which is ambiguous. Toxicity depends on the type of tested GBNs and the type of used cells; maximum non-lethal doses range from 75 µg/mL to 1000 µg/mL [71]. As mentioned above, 2D-NMs include also nanoclays. A variety of nanoclay materials are competitors of GBNs [72,73,74]. A comparative study of the cytotoxicity of kaolinite, halloysite, and carbon-based NMs showed that carbon-based NMs are the most toxic and genotoxic to cells. GO significantly increased the fraction of apoptotic cells and was the most cytotoxic and genotoxic nanomaterial. No significant effect of the shape of the tested NMs on their internalization and cytotoxicity was demonstrated [75]. In this context, it is logical that the joint application of GO and kaolin has been found to reduce the negative effects of GBNs (by almost 20%, most likely because of coagulation of the NPs with each other) [76,77].

In this brief up-to-date overview, attention is paid to the applications of GBNs, i.e., GR, graphene quantum dots (GRQDs), GO, graphene oxide quantum dots (GOQDs), and rGO. These materials as well as their various modifications and combinations with other compounds will be discussed in their medical–-pharmaceutical and agroecological applications, i.e., as materials investigated for their antineoplastic and anti-invasive effects, which are effects against various plant pathogens, and as carriers of bioactive agents (drugs, pesticides, and fertilizers). The negative effects on living organisms, including their mode of action are also briefly analyzed. Methods for the preparation and characterization of 2D graphene-based nanomaterials are not given, as they can be found in detail in numerous papers, e.g., [7,10,35,78,79,80,81,82].

## 2. GBNs as Drugs and Nanocarriers

GBNs are used for their intrinsic activity as anticancer or antimicrobial agents and for their properties as drug nanocarriers. In the sections mentioned below, the effects of the GBNs themselves are always mentioned first, followed by variously modified GBNs, using various organic or metallic compounds.

### 2.1. GRs and GRQDs

Characterized with an ultrathin, layered structure and high surface-to-volume ratio, enabling high loading of various therapeutics, 2D-NMs sustain a prolonged release at the target site, whereby some 2D nanocarriers can provide on-demand therapeutic release, as a response to external stimuli. For medicinal use, the 2D nanocarriers, which are biocompatible and degrade into nontoxic products, are favorable [83]. These 2D materials possess important properties, such as semiconductivity, high surface area, a chemical nature suitable to be functionalized or decorated, and a stable structure and targeting ability, which can ensure a controlled and sustained release of drugs; these materials can also be used for thermal-based therapies, which predestine them to be applied in cancer therapy [84]. Recent progress in the surface modification of GR and its derivatives, appropriate for drug delivery systems, was summarized by Jonoush et al. [85]. GR, similarly to other carbon-based materials, such as carbon nanotubes, carbon quantum dots, or fullerenes, can be used as effective nanocarriers of radiopharmaceuticals in cancer therapy or as theranostic systems [86]. In addition, GR has emerged as a potential anticancer agent and has shown great potential in targeting tumor mitochondria in a safe and targeted fashion. Tabish and Narayan [87] overviewed strategies for fabrication of mitochondria-targeted GR, for targeted destruction of cancer cells, and discussed recent progress in the application of mitochondria-targeted GR in chemotherapy, PDT, PTT, and combination therapies. GR-based nanomaterials, showing potential to be also used in fighting the most challenging viruses and immunogenic disorders, were overviewed by Ebrahimi, et al. [88], and the latest progress in the use of GR nanofilled composites in dental applications was presented by Alkatheeri [89]. Begum et al. [90] discussed, in their review paper, 2D materials (GO, 2D transition metal dichalcogenides, 2D MXenes, and 2D heterostructure materials) suitable to be used as antimicrobial materials, including criteria for developing novel antimicrobial 2D and heterostructure materials, suitable to eliminate bacterial infections. Water-soluble 2D nanosheets, containing certain functional groups (carboxylic and hydroxy), fabricated by Pandit and De [91], showing a layered structure with a thickness about 1.2 nm and zeta potential of −38 ± 2.5 mV, were able to efficiently mimic the NADH peroxidase-like activity [92] and can, selectively, bind to the active sites of the enzyme via a competitive pathway and catalyze the oxidation of NADH to NAD^+^ and dopamine to aminochrome, in the presence of H_2_O_2_. In the control experiment, in the absence of 2D nanosheets, only very low oxidation of NADH was observed in the presence of H_2_O_2_.

Electro-mechanical properties, high surface area, great loading capacity, and elevated thermal capacity of GR nanoribbons predestine them to be used in bio-imaging, green chemistry, material sciences, novel drug delivery, etc. Moreover, functionalized GR nanoribbons are characterized with improved adsorption and adhesive-binding properties to mammalian cells, and, therefore, can be used as a bio-carrier for gene-transfect-ion and nucleic-acid delivery [93]. A functionalized GR-dendrimeric system, designed via Fe_3_O_4_ nanoparticles (NPs) and β-cyclodextrin, modified by NH_2_ groups and grafted with GO, was used for co-delivery of encapsulated melatonin and DOX. The nanoformulation exhibited good anticancer performance, and a synergistic antitumor effect against the human osteosarcoma cell lines, including Saos-2 and MG-63, likely due to down-regulation of X-linked inhibitor of apoptosis (XIAP), survivin, and human telomerase catalytic subunit (hTERT) (*p* < 0.0001), which reduced toxicity in normal cells [94]. pH-sensitive BSA-stabilized GR/chitosan (CS) nanocomposites, encapsulating DOX, were tested for drug release at pH 7.4 and pH 4.5, for one month. The presence of bovine serum albumin (BSA) in nanocomposite pronouncedly reduced the burst release observed in CS NPs nanocomposite and nanocomposite containing 2 wt% of GR released 84% of DOX in 28 days, with uniform release in the first 24 h; the released drug from the nanocomposite greatly inhibited the proliferation of SK-BR-3 breast cancer cells at acidic pH [95]. It should also be noted that antibacterial GR-based hydroxyapatite/CS coating with gentamicin exhibited considerable antibacterial effect against *Staphylococcus aureus*, causing a reduction in viable cells with >3 logarithmic units, while in *Escherichia coli* it showed a bacteriostatic effect, reducing the number of viable cells <3 logarithmic units. Due to its good biocompatibility, this coating has the potential to be used in bone-tissue engineering, as a hard-tissue implant [96]. Using poly(vinyl alcohol) (PVA) as a capping agent, Das Jana et al. [97] designed a highly transparent coating, based on Cu−GR nanocomposite, able to exhibit high antiviral activity in the solid form, which could be implemented on various surfaces, to inhibit the transmission of respiratory virus infections.

**Figure 4:**
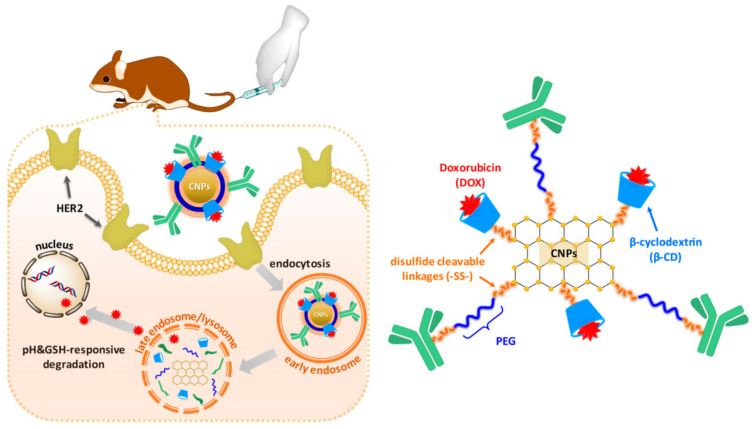
Illustration of cellular uptake and drug release of DOX-loaded dual stimuli-responsive degradable GRQDs, in HER2-positive breast cancer cells. Adapted from [103], copyright 2020, MDPI.

Recent progress in the fabrication of GRQDs used in bio-sensing, bio-imaging, drug delivery, anti-bacterial activity, and PTT/PDT, as well as in optoelectronic applications, was summarized by Kadian et al. [98]. A smart dressing for the theranostics of diabetic wounds was fabricated using GRQDs-decorated luminescent porous silicon, which was loaded with peptide drugs beneficial for rapid diabetic wound healing (insulin and epidermal growth factor (EGF)) and embedded in CS film. The dressing showed stimuli-responsive drug release, under slightly acidic and highly oxidative conditions in diabetic wounds, and enhanced the proliferation and migration of cells, along with considerable healing of diabetic wounds [99]. The cellular uptake of GRQDs, loaded into layered double hydroxide (LDH) NPs by rat bone marrow derived mesenchymal stem cells (MSCs), increased the endocytosis and phenotypic transition of macrophage in LDH-GRQD nanocomposites and was easier, due to inflammatory regulation of LDH, suggesting therapeutic potential of such nanocomposites to be used in bone defects regeneration [100]. Polyethylene glycol (PEG)-functionalized GRQDs-based magnetic NPs, which can function as a superb delivery system, for the controlled release of anticancer drugs as well as imaging agents for cancer cells [101], plus targeted curcumine (CUR) delivery and cancer cell imaging were also observed, using CUR-loaded GRQDs decorated with hyaluronic acid, which were adsorbed on HeLa cells, unlike L929 cells, and greatly inhibited HeLa cell viability [102]. PEG-GRQDs, conjugated with herceptin (HER) and DOX-loaded β-cyclodextrin, were designed by Ko et al. [103], for the treatment of human epidermal growth-factor receptor 2 (HER2)-positive breast cancer. HER, cyclodextrin, and GRQDs are interconnected by disulfide bridges. High levels of glutathione and low pH in cancer cells hydrolyze disulfide bonds and cause the controlled release of DOX from cyclodextrins, see Figure 4. The described system showed high cellular uptake, high in vitro and in vivo efficacy, and low toxicity. Glucosamine-conjugated GRQDs, showing a size of 20–30 nm and <10 layers, releasing after 150 h 37% and 17% of encapsulated CUR at pH 5.5 and pH 7.4, respectively, showed higher cellular internalization via glucosamine-receptor-mediated endocytosis in breast cancer cells as well as pronouncedly higher cytotoxicity against MCF-7 cells, compared to CUR/GRQDs [104]. Tryptophan-functionalized GRQDs were also characterized with high CUR-loading capacity and pH-sensitivity [105]. GRQD cross-linked carboxymethyl cellulose exhibited pH-sensitive swelling and biocompatibility as well as showed pH-sensitive oral delivery of encapsulated DOX; cytotoxicity studies using human colon adenocarcinoma HT29 cells suggested the prepared hydrogel can be used as a pH-triggered site-specific drug delivery system [106]. NH_2_-functionalized and nitrogen-doped GQDs can be used as photosensitizer in PDT and generate more reactive oxygen species (ROS) than conventional GQDs, under short (60 s) low-energy irradiation, enabling the complete elimination of a multidrug-resistant strain of methicillin-resistant *Staphylococcus aureus* (MRSA), a Gram-positive bacterium. Moreover, they were characterized by high photostability and can also be applied as contrast probes, for use in biomedical imaging. CUR-loaded GQDs exhibited a remarkable increase in ROS production and, under irradiance with blue-light (405 nm; 30 J/cm^2^), and a dose of 100 μM, achieved enhanced colony-forming-unit (CFU) reduction, approximately 3.5 log_10_ against *Pseudomonas aeruginosa*, MRSA, *E*. *coli*, and *Candida albicans*, suggesting that the antimicrobial photodynamic effects of CUR-loaded GQDs have the potential to be used for the treatment of resistant infections [107]. Water-soluble nanoconjugates of GRQD and boron-dipyrromethene-dye derivatives are able to generate great amounts of ^3^O_2_ and ^1^O_2_, which exhibit excellent water solubility and high PDT efficiency (IC_50_ value of 30 nM), were able to enhance the local cellular concentration of the conjugated photosensitizer and caused apoptotic death of MDA-MB-231 cells [108]. The pH/ultrasound-responsive nanocarriers, based on magnetic core-shell ZnFe_2_O_4_@mesoporous ZnO@GQDs and ZnFe_2_O_4_@mesoporous ZnO@nitrogen doped GRQDs nanocarriers loaded with CUR, exhibited controlled and targeted drug release, by using pH adjustment and ultrasound irradiation [109].

Ji et al. [110], using molecular dynamics simulations, studied the capability of GR and GO to deliver drugs with different molecular size and polarity, such as primidone, pregabalin, and bortezomib, and found that, due to the electrostatic interactions or hydrogen bonding, GO exhibits higher adsorption intensity than GR, resulting in a more difficult release of the adsorbed drugs from GO, after entering the membrane; the loading and unloading of primidone by both GR and GO, was more effective compared to pregabalin and bortezomib. Whereas GRQDs and GO did not show cytotoxicity against Kerman male breast cancer/71 (KMBC/71) and MCF-7 tumor cells, their CUR-loaded formulations killed >50% of tumor cells and exhibited a synergistic effect on the anti-tumor activity of CUR. However, while exposure to GRQDs-CUR resulted in cell death of the majority of KMBC/71 mammospheres (99%), at application of GO-CUR, cell death was observed in only 21% of MCF-7 mammosphere cells; the expression pattern of miR-21, miR-29a, and Bax/Bcl-2 ratio in KMBC/71 and MCF-7 mammospheres, upon treatment with GO-CUR and GRQDs-CUR, was different as well [111].

### 2.2. GO and GOQDs

As a promising nontoxic nanocarrier for the delivery of CUR, BSA-modified nanoscale GO-like carbon-based NPs, exhibiting pH sensitivity as well as controlled-drug-release and antiproliferative ability against MCF-7 breast cancer cells, were recommended by Danafar et al. [112]. GO as a carrier for the delivery of methotrexate was tested by Abdelhamid and Hussein [113]. GO sheets, fabricated by Mohanta et al. [114] with zeta potential of −9.3 mV and lateral thickness ca. 6.45 nm, showed H_2_O_2_ scavenging activity (IC_50_ 61.91 ± 1.14 μg/mL), whereby their 2,2-diphenyl-1-picrylhydrazyl (DPPH) and H_2_O_2_ scavenging activity showed a dose-dependent increase in the concentration range 25–400 μg/mL. GO sheets exhibit antioxidant activity, whereby GO is a weak hydrogen donor, due to the non-phenolic nature of most OH groups on GO, which reside at basal sp^3^-carbon sites. The cytotoxicity of GO sheets against the HaCaT normal cell line was minor, while it was remarkable and dose-dependent against the human breast cancer MDA-MB-231 cells. It should be noted here that, although GBNs are generally considered to cause oxidative stress [40,42,115], in some cases they are able to scavenge radicals [116]. The antioxidant activity of GBN lies in the scavenging of hydroxyl and superoxide radicals. In was observed that few-/multi-layer GR is more active than GO, despite its lower surface area, suggesting that the main scavenging activity is associated with pristine sp^2^ carbon domains on basal surfaces, rather than with H-donation from hydroxyl or hydroquinone groups [117,118]. Thus, GBNs can protect various biomolecular target molecules from oxidation.

GO exhibited a protective impact against the Rubella virus infection of human lung epithelial carcinoma cells (A549), human chondrocyte cells (TC28a2), and lowered cytopathic changes caused by the virus to human cells [119]. Pulingam et al. [120] summarized the findings related to bactericidal mechanistic actions of GO and highlighted roles of physicochemical factors such as size, aggregation, functionalization, and adsorption behavior, affecting its antibacterial properties. A co-culture of *E. coli*, with 8 μg/mL GO for 2 h, resulted in up to 90% inactivation of bacterial cells, via producing ROS and inactivating superoxide dismutase (SOD) and catalase (CAT) enzymes [121].

Functionalized GO, characterized with remarkable adjuvant activity in activating cellular and humoral immunity, can serve as a vaccine carrier [122]. Gelatin hydrogels doubly cross-linked with GO and glutaraldehyde-encapsulating *Kluyveromyces lactis* showed effective cell entrapment and proliferation of this probiotic, tunable degradation rates, pH-dependent swelling ratio, and ensured stability of formulation in simulated gastrointestinal (GI) media [123]. Bacterial-cell survival in starvation conditions in the presence of GO is due to the fact that the oxygen-containing functional groups of GO are similar to the molecular structure of methylglyoxal, which bacteria produce to adapt to nutrient imbalances, and is detoxified by glyoxalase enzymes. Hence, GO can be considered as a methylglyoxal-mimicking nanomaterial, enabling rearrangement of cellular metabolism and defenses [124]. Pristine GO exhibited toxicity against *E. coli* and *S. aureus,* however, the antibacterial effect of GO was neutralized after its functionalization with octadecylamine [125]. The combination of functionalized GO and AGXX^®^, coated on cellulose fibers, inhibited the growth of MRSA strain *S. aureus* 04-02981 by 99.98%, repressed genes related to biofilm formation and virulence (such as *agr*, *sarA*, and *SaeRS*) as well as genes crucial for survival in biofilms (such as arginine metabolism arc genes); induced the expression of siderophore biosynthesis genes (*sbn*); and affected expression of genes associated with K^+^ transport, intracellular survival, and pathogenesis (*kdp*). Hence, this material could be applied in antimicrobial surface coatings [126]. Benzofurazan-derivatives-modified GO nanocomposite can inhibit bacterial (*S. aureus*, *E. coli*, and *P. aeruginosa*) biofilm formation and induce cytotoxicity in the human colon cancer HCT-116 cell line, along with limited impact effects on normal human BJ fibroblasts [127]. A considerable antiangiogenic effect of GO in primary human endothelial Huvec cells was due to the combination of the physical hindrance of internalized GO aggregates, induction of oxidative stress, and alteration of some metabolic pathways. Besides, steric hindrance of GO intracellular aggregates, perturbing the correct assembly of cytoskeleton and distribution of mitochondria and causing impairment of cell migration, affected the formation of capillary-like structures and the consumption of niacinamide, which is a precursor of energy carriers, as well as several amino acids involved in the regulation of angiogenesis [128].

The in vivo tumor uptake of nanoscale sheets of carboxylated GO (40 nm), radiolabeled with ^99m^Tc and radiolabeling yield of 97.3 ± 0.45% in a tumor-bearing mice model was high, indicating their promising potential to be used as an imaging agent [129]. On the other hand, carboxylated GO nanosheets decorated with ZnO NPs and post-functionalized with Pluronic^®^ demonstrated selectivity toward U87MG and U138MG human glioblastoma cell lines, but they were less cytotoxic than free ZnO and did not interfere with the mechanisms for inducing apoptotic pathways. However, the nanocomposite induced changes in adhesion points as well as roughness of the tumor cell membrane and was taken up through vesicles and accumulated in the nucleus, which can induce cell death by apoptosis [130].

Mannose-decorated CS-functionalized GO nanocarrier, loaded with sulfated polysaccharide ulvan from green macroalgae as a model anticancer drug, achieved drug entrapment efficiency of 88% and showed a pH-dependent-controlled drug release and targeted drug delivery in human glioblastoma cell line (U87) in vitro [131]. PEG functionalized GO nanocarrier is characterized with high delivery efficiency and controlled release of chemotherapeutics, anticancer drugs, and bioimaging agents as well as with high near-infrared (NIR) absorbance and capacity in photothermal treatment. An accumulative release rate of 71.12% in 12 h, under slightly acidic condition, and considerable inhibitory effects on rat gloom cells were observed with temozolomide-loaded PEGylated GO [132]. Application of NIR radiation can modulate the phototoxicity of PEGylated GO NPs, have an effect on highly invasive Colon26 colon cancer cells as well as low invasive HT29 colon cancer cells, and be biocompatible with normal cells (little or no DNA damage, no mitochondrial effects) [133]. PEGylated nanoscale GO (324.6 nm; zeta potential from −32.9 to −21.6 mV), showing a wrinkled surface of the nanosheets, when combined with application of NIR irradiation laser (5 min; 1.5 W/cm^2^), causes growth inhibition of low invasive colon cancer cells (HT29) and exhibits wound-closure ability, suggesting that it can be used as a smart nanocarrier in colon-cancer-targeted therapy [134].

GO nanoribbons, functionalized with folic acid (FA), which were loaded with a selective estrogen receptor modulator, raloxifene hydrochloride, showed multi-layered structure and entrapment efficiency (EE) of 56%, exhibited a pH-dependent drug release as well as dose- and time-dependent cytotoxic effects on MCF-7 and MDA-MB-231 breast cancer cells, and their cellular uptake, by both cancer cell lines, was considerably higher than that of non-functionalized oxidized GR nanoribbons [135]. Nanocarriers prepared using GO functionalized with polymers (polyethyleneimine, PEG, and CS) and FA, which were loaded with Pt anticancer drugs, including cisplatin, carboplatin, oxaliplatin, and eptaplatin, showed an enhanced cumulative release rate of drugs (>60%) in an acid environment compared to a neutral one, and were characterized with low cytotoxicity with cell-viability rates >80%, inhibiting the growth of the SKOV3 cell line in vitro [136]. GO loaded with protocatechuic acid (PCA) and chlorogenic acid (CA) exhibited anticancer activities in both passive and active targeting, induced late apoptosis in HepG2 cells and cell cycle at the G_2_/M phase, and caused depolarization of mitochondrial membrane potential as well as an upregulation of ROS, when HepG2 cells were induced by nanocomposites. At exposure of HepG2 cells to GO−PCA/CA−FA, the dual drug nanocomposite exhibited considerable anticancer activity but less toxicity than at the application of pristine drugs or GO−PCA/CA nanocomposite, likely due to the presence of FA [137].

Paclitaxel (PTX) and CUR drugs were loaded into a nanocarrier consisting of GO-grafted poly(epichlorohydrin) and OH groups that were grafted with hyperbranched polyglycerol and encapsulated into pullulan nanofibers using electrospinning, which exhibited sustained release in a medium with pH 7.4, and this drug delivery system has the potential to be used for local chemotherapeutic applications [138]. DOX-loaded Pluronic^®^ F127/GO nanohybride induced a higher apoptosis rate of U251 cells than that of free DOX (12.27 ± 0.06% vs. 8.20 ± 0.06%), affected the mitogen-activated protein kinase (MAPK) signaling pathway, and induced the intrinsic pathway, of apoptosis for the activation of caspase-3 in U251 cells [139].

Using triphenylphosphine linkage to decorate the surface of GO nanosheets by AgNPs, the surface of GO sheets was covered with AgNPs via non-covalent and permanent bonding, altering structural and electronic properties as well as inducing oxidative stress, resulting in death of *Bacillus subtilis*, *Enterococcus faecalis*, MRSA, *S. aureus*, *E. coli*, *Serratia marcescens*, *Shigella* sp., *Salmonella* sp., *Serratia liquefaciens*, *Proteus* sp., *Enterobacter cloacae*, and *P. aeruginosa* [140]. By co-incorporation of high amounts of GO/AgNPs into poly-l-lactic acid (PLLA) fibrous deposited on Mg alloy via electrospinning, improved antibacterial performance against *E. coli* and *S. aureus* was observed, compared to that of Mg alloy and neat PLLA fibrous, and the coating also showed the adequate corrosion resistance and cytocompatibility required for use in orthopedic applications [141]. GO−Cu nanocomposites were reported to suppress cariogenic *Streptococcus mutans* biofilm formation, and already a dose of 10 μg/mL GO−Cu altered the biofilm architecture as well as damaged the production and distribution of exopolysaccharides and dysregulated the expression of exopolysaccharide-associated genes but exhibited only minimal cytotoxicity [142]. GO-modified porous TiO_2_ coatings exposed to 808-nm light irradiation exhibited superb antibacterial activity against *S. mutans* caused by the synergistic effects of hyperthermia and generated ROS; they could be used in clinical applications to combat implant-associated infections [143]. Ternary nanocomposites based on GOQDs, polyaniline, and manganese oxides exposed to photoirradiation at 365 nm, exhibited antimicrobial activity toward both *E. coli* and *S. aureus*, primarily due to the photocatalytic generation of ·OH radicals and photogenerated holes, inducing oxidative stress in bacterial cells, whereby nanocomposites containing mainly Mn^3+^ component were less active than those with high Mn^2+^ and Mn^4+^ content [144].

AuNPs-decorated GO nanocomposites, GO−Au and GO−Au (×2), considerably increased cell viability of MSCs, showed good antioxidative activity, sponged the immune response toward monocyte-macrophage transition, and suppressed the activity of platelets. In addition, they increased cell motility and differentiation of various MSCs-derived cell types (e.g., neuron cells, adipocytes, osteocytes, and endothelial cells), reduced induction of fibrotic formation and M1 macrophage polarization, while higher induction of M2 macrophage and stimulation of the endothelialization was observed in Au-deposited GO nanocomposites implanted in an animal model, suggesting their superb immune compatibility and anti-inflammatory impact in vivo and in vitro [145]. GO conjugated with Au@Ag and Fe_3_O_4_ NPs, facilitating it with surface-enhanced Raman scattering spectroscopic (SERS) tracking and magnetic targeting abilities, was able to covalently bind to the anti-HER2 antibody, enabling both active and passive targeting of SKBR3 cells (human breast cancer cells expressed with HER2), was used for co-delivery of DOX and 9-aminoacridine. The nanocarriers were internalized into the lysosomes and exhibited pH-responsive drug release in acidic environment, showed enhanced cancer cytotoxicity compared with nanocarriers loaded with a single drug, and increased cytotoxicity against cancer cells, which was observed even with relatively low concentrations of the drugs [146]. Nanocomposites fabricated by functionalization of GO with polyvinylpyrrolidone (PVP) and then grafted with Fe_3_O_4_ NPs loaded with quercetin exhibited pH-responsive controlled drug release and biocompatibility to non-tumorigenic epithelial HEK 293T cells, with higher toxicity to MDA MB 231 human breast cancer cells than free quercetin. Moreover, targeted drug delivery using this magnetic GO nanocomposite can be controlled by an external magnetic field [147]. Fe_3_O_4_−GO nanohybrids coated with β-cyclodextrin−cholic acid−hyaluronic acid polymer, characterized with multiple-targeted features (the cholic acid supplied the hepatic target, CD44-receptor target of hyaluronic acid, and magnetic target of Fe_3_O_4_), exhibited local chemo-photothermal synergistic effects via directly generated apoptosis of hepatocellular carcinoma cells, trigging the release of encapsulated camptothecin, resulting in a tumor inhibition rate >90%; this nanohybrid could be used for enhanced liver tumor therapy [148]. Then, 5-fluorouracil (5-Fu)-loaded super-paramagnetic iron oxide (SPION) NPs/GO particles, coated with polycaprolactone (PCL)/CS copolymers injected intravenously with subsequent application of magnetic field and exposure to an alternating magnetic field (AMF) (40 A/m, 13.56 MHz), enhanced the tumor site temperature to 43 °C and considerably reduced the plating efficiency of the cells, while increasing the Bax/Bcl-2 ratio and reflecting cell susceptibility to apoptosis; in vivo reduced the growth of CT-26 tumor cells and increased the life span of the tumor-bearing mice, compared to that of the free 5-Fu drug [149]. Magnetic γ-Fe_2_O_3_ grafted to the surface of GO and, subsequently, covalently bound with mitochondrion targeting peptide (MitP), released encapsulated mitoxantrone (MTX) at exposure to an alternating magnetic field (AMF). The MTX-loaded GOMNP-MitP released MTX to the mitochondria, resulting in strong impairment of mitochondrial functions, reflected in suppression of ATP production and reduction in mitochondrial membrane potential, which, ultimately, activated apoptosis [150]. Fe_3_O_4_@PEG-coated triazine dendrimer modified GO nanocomposite, showing mean thickness of the nanosheets of ca. 144.21 nm exhibited controlled pH responsive release of encapsulated DOX, was not cytotoxic and showed superb biocompatibility, and exhibited higher cellular uptake within 4 h and higher apoptotic effects against MCF-7 cancer cells than free DOX [151]. The presence of Pb^2+^ was found to reduce cytotoxicity of GO nanosheets against A549 cells; it suppressed phospholipid extraction and diminished the oxidative stress, nutrient depletion, and sheet adhesion of GO [152]. The effects of selected nanomaterials are summarized below.

### 2.3. rGO

**Figure 5:**
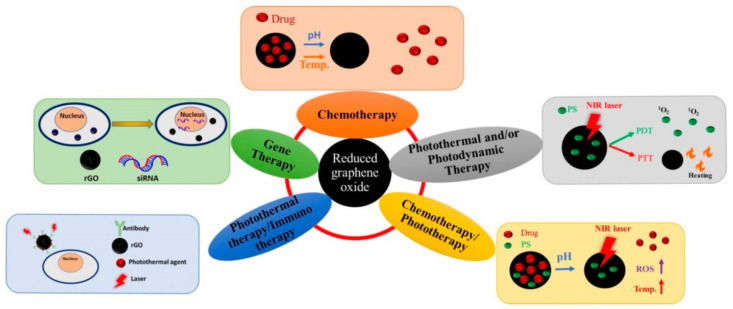
Applications of reduced graphene oxide in therapy of cancer. Adapted from [163], copyright 2021, MDPI.

Taylor et al. [153] summarized the findings related to drug release kinetics of DOX-loaded GO and rGO for ovarian and breast cancer therapeutics. As stimuli for drug release changes in pH, NIR or an ultrasound were used, and in most cases the best fit with experimental data was obtained with the Weibull kinetic model. The researchers also stated that computational modelling performed prior to pre-clinical testing can contribute to design-controlled and sustained DOX release systems, suitable for therapeutical application. Metal-free antibacterial additives for cotton fabrics showing resistance to detergent washing treatments were designed by Biagiotti et al. [154], using salicylic acid-functionalized GO and rGO. Electrospun nanofibrous composite membranes prepared using citric acid-functionalized CS containing 0.250 wt% rGO-tetraethylene pentamine (TEPA) characterized with good cytocompatibility, highest level of cell development and proliferation, and good anti-biofilm activity against *P. aeruginosa* and *S. aureus* are suitable to be applied in wound dressings [155]. Smart and pH-sensitive rGO/arabinoxylan/CS composite showing controlled release of antibacterial drug silver sulfadiazine, which can be used for wound dressing, was designed by Khan et al. [156]. Buprenorphine-loaded Pluronic^®^ F127-rGO transdermal (noninvasive) hydrogel, showing prolonged release up to 14 days and an analgesic effect, could be used to manage chronic pain in osteoarthritis [157]. Using Pluronic^®^ F68-rGO hydrogel loaded with lidocaine exhibiting prolonged drug release (up to 10 h), an anesthetic effect in the radiant heat tail flick test and sciatic nerve block model as well as the prolongation of effects of local anesthesia can be achieved [158]. Composite nanocarriers combining Au nanorods, partially reduced GO with chlorin e6 photosensitizer, and tumor targeting ligand FA designed for NIR-induced synergistic PDT/PTT and photoacoustic (PA) imaging, which were characterized with remarkably improved generation kinetics of ^1^O_2_ and high photothermal conversion efficiency, were internalized in MCF-7 cancer cells overexpressing folate receptor, and destroyed 95% of cancer cells by irradiating simultaneously with 670 nm and 880 nm lasers for 5 min [159]. AgNPs/rGO composites formed of micrometer-sized rGO sheets decorated by AgNPs of ca. 70 nm showed antimicrobial and photoantimicrobial activities. Inactivation of *S. aureus*, under irradiation with blue light, can result from (i) chemical effect stimulated by the release of Ag^+^ ions from AgNPs; (ii) photocatalytic activity induced by AgNPs/rGO composites, increasing photoinactivation of bacteria via the excited-plasmons of the AgNPs when anchored on rGO; and (iii) photodynamic effect caused by bacterial endogenous photosensitizers at exposure to blue-light irradiation [160]. Nanocomposite of Zn-doped hydroxyapatite with rGO showed 3.4-fold higher antibacterial properties compared to pure hydroxyapatite NPs and pronouncedly, enhanced alkaline phosphatase activity and proliferation of mesenchymal stem cells, suggesting its suitability to be used in bone-tissue engineering [161]. Catechin grafted in rGO/ZnO nanocomposite showing hexagonal wurtzite structure with aggregated morphology and size of 111.7 nm, exhibiting enhanced drug release at acidic pH within 24 h, showed a dose-dependent antiproliferative effect of Cargo/ZnO and generated high ROS levels, resulting in cell-membrane damage and enhanced cytochrome C release, and ultimately also in apoptosis. This nanocomposite also showed strong reduction in biofilm formation (IC_50_ 5 ± 0.25 μg/mL), and disrupted biofilm architecture consisting of reduced microcolonies, mostly dead cells. The nanocomposite would be suitable for treatment of infectious disease and lung cancer [162]. The effects of selected nanomaterials are summarized in Table 1. In addition, rGO applications in cancer therapy are shown in Figure 5.

## 3. Impact of GBNs on Harmful Insects

As already mentioned, GBNs are also being investigated as a useful tool in fighting insect pests. The activity of the GBNs themselves was investigated in several models, and interesting facts were found. Exposure of lepidopteran insect *Ostrinia furnacalis* to GO stimulated growth of insects, activated trypsin-like serine protease, glutathione *S*-transferase, heat shock protein, and glycosyltransferase; trypsin gene was evaluated as one of the crucial genes responsible, for accelerating growth of insects fed with a GO diet. In addition, higher levels of cholesterol, triacylglycerides, and lipids were observed in insects exposed to GO [164]. Flasz et al. [165] investigated impact of multigenerational intoxication with GO supplemented in food using an *Acheta domesticus* model insect and found that chronic GO intoxication negatively affected expression pattern of vitellogenin (VTG), which is important in embryo nutrition. In contrast to low VTG expression observed in the 1st generation of *A. domesticus* insects, the 2nd generation exhibited high VTG expression, and in the 3rd generation the VTG expression was balanced, suggesting that GO-induced stress got under control. According to researchers, the chronic GO intoxication might impair the regular formation of the VTG quaternary structure, with a negative impact on the developing embryo. However, involvement of the epigenetic mechanisms in the information transfer to the next generations, related to the response to this risk factor, might contribute to ensuring a high rate of reproduction. Dziewiecka et al. [166] observed considerable differences in the life cycle and reproductive processes of A. *domesticus* exposed to 0.2 μg, 2 μg, and 20 μg GO per gram of food for three generations, which were not always dose-dependent. Whereas the most unfavorable impact of GO on studied characteristics (activity of antioxidant enzymes, level of apoptosis, hatching abilities, body mass and length of insects, and their survival rate) was observed in the 2nd generation of insects exposed to GO, an increase in DNA damage was observed only in the 3rd generation. House cricket females fed with low dietary doses of GO (20 μg/g food) and GO−AgNPs, (20:400 μg/g food) for 10 days exhibited time-dependent changes in food (energy) consumption and utilization. Considerably reduced consumption and assimilation was observed on the beginning of the experiment (0–3 days) but, later, compensation mechanisms were triggered, resulting in a minor drop in consumption and assimilation during days 3–6, compared to control; in days 6–10, the consumption and assimilation, along with the activities of most gut enzymes, achieved the values observed in the control insects. In addition, insects exposed to GO−AgNPs composite possessed higher content of body water, indicating its improved uptake [167]. Exposure of the ovary cell line of *Bombyx mori* to >25 mg/L GO induced oxidative stress, ROS accumulation, and DNA damage in cells as well as pronouncedly diminished their survival, while female *B. mori* larvae fed with mulberry leaves, which were treated with 25 mg/L GONPs, considerably reduced their gonadosomatic index and enhanced oxidation stress and antioxidant enzyme activity in ovary tissues; numbers of oogonia and oocytes in ovarian tissues were reduced, while formation of peroxisome and vacuoles in follicle cells showed an increase, reducing the transcription of genes related to ovarian development in *B. mori* (*Vg*, *Ovo*, *Sxl-s*, *Sxl-l*, and *Otu*) and lowering the amount of spawning, suggesting a toxic impact by GO on reproduction [168]. A diet containing 1000 μg GO per gram of diet dry mass considerably reduced the fecundity and fertility of *Spodoptera frugiperda* as well as the efficiency of food conversion into biomass, and maximal approximate digestibility in the larvae showed a decrease as well [169].

GBNs, on the other hand, serve as carriers and stabilizers for other insecticides, and they are able to potentiate their activity. A 25 kDa cysteine protease extracted from seeds of *Albizia procera* (ApCP), showing insecticidal activity, which was encapsulated with GRQDs and applied at a dose 7.0 mg of ApCP per a gram of wheat flour and grains, reduced number of eggs and larvae of *Tribolium castaneum* (Herbst), by 49% and 86%, respectively, and showed improved insecticidal activity compared to free ApCP, which was manifested by 98% reduction in adult eclosion and 72% larval mortality, and even better results have been achieved at treatment of stored grain insect pest *Rhyzopertha dominica* (Fabricius) with the same dose of GRQDs-encapsulated ApCP (reduction in eggs and larvae and eclosion by 72%, 92%, and 97%, respectively, and an increase in larval mortality to 90%) [170].

Composites of tetradecenyl acetate pheromones with GO and NH_2_-GO, in which pheromones were assembled into a multilayer, were found to extend the diffusion path in pheromone traps via stimulating electrophysiological response in the antenna, leading to considerably enhanced efficiency in trapping of *Tuta absoluta* insects due to the extension of the pheromone life, compared to the commercial septa [171].

Loading of PEGylated-GO with emamectin benzoate insecticide remarkably improved aqueous solubility of the insecticide; the formulation responded to pH stimuli, ensured sustained release of pesticide, and showed enhance resistance to stress induced by UV light up to t_1_/_2_ of 521.16 h, as well as sustainable insecticidal activity [172]. CS–GO nanocomposites applied at concentrations 1%, 2%, and 3% (*w*/*w*), which were tested as water-solubilizing agents for rotenone insecticide, increased rotenone aqueous solubility by 34.40%, 38.80%, and 46.30%, respectively, thereby increasing its bioavailability. In the adsorption of rotenone on CS−GO nanocomposites having −OH, −COOH and −NH functional groups, hydrogen bonding, and π−π interaction played a crucial role [173]. Synergistic effects of GO against *Tetranychus cinnabarinus* observed with GO−acaricide (avermectin, bifenazate, etoxazole and spirodiclofen) nanocomposites were reported by Zhou et al. [174]. Binding of GO to cuticle protein (CPR), along with suppression of the CPR gene resulted in increased permeability of insect cuticule, enabling increased efficiency of tested acaricides. Similar results were obtained with avermectin, bifenazate, etoxazole, and spirodiclofen-loaded GO nanoheets, which were found to adsorb and damage the cuticle of *T. cinnabarinus* spider mites via binding to a CPR, and upregulate expression of the CPR gene, resulting in enhanced cuticle permeability of the insect, which considerably contributed to the improved efficiency of acaricides. Moreover, dehydration and disturbed construction of the cuticle layer was observed due to silencing of the CPR gene by iRNA [174]. GO mixtures with pyridaben, chlorpyrifos, and β-cyfluthrin acaricides showed 1.77-, 1.56-, and 1.55-fold higher contact toxicity against *Tetranychus truncatus*, and 1.50-, 1.75-, and 1.78-fold higher contact toxicity against *T. urticae*. The amelioration of the efficiency of acaricides against spider mites is due to their adsorption on the surface of GO, which functioned as a carrier [175]. Gao et al. [176] combined cyhalothrin, bifenthrin, and fenpropathrin with GO to prepare controlled release nanopesticides with excellent stability. The matrix released all of these pyrethroids as a function of temperature, and nanocomposites, which were shown to have much higher biological activity than individual pesticides against *T. urticae* Koch, indoors and in the field. GO pesticides were adsorbed on the cuticle of *T. urticae* as well as highly evenly on the surface of the bean leaves. GO mixtures with β-cyfluthrin, monosultap and imidacloprid showed 2.1-, 1.51- and 1.83-fold higher contact toxicities to *Ostrinia furnacalis* compared to application of the individual insecticides; synergistic impact was due to physical damaging of insect cement layer and subsequent strong water loss and formation of new channel by a disrupted cement layer, enabling penetration of insecticides [177]. GO-binary mixtures with malathion (ML) and endosulfan (EN) insecticides (1:1 and 1:2) exhibited toxic impact on *Aedes aegypti.* Compared to the application of pure insecticides, the toxicity of GO-binary mixture with ML was higher by 80.43% and GO-binary mixtures with EN achieved even a 6.43-fold higher toxicity. In larvae exposed to GO−ML mixtures cuticular deposition of black soot was observed, while exposure of larvae to GO−EN resulted in disintegrated gut viscera. Irritant potential of tested mixtures of GO with insecticides was also estimated [178]. Selected applications for harmful insects are summarized in Table 2.

## 4. Applications against Plant Patogenic Microorganisms

Exposure of *Ralstonia solanacearum* to GO caused damage to bacterial cell membrane, reduced ATP levels and considerably enhanced malondialdehyde (MDA) levels, suggesting oxidation of lipids in bacteria. In addition, with the exception of *popA*, the expression levels of genes involved in virulence and motility, *phcA*, *hrpB*, and *flgG*, were pronouncedly downregulated, while oxidative stress genes, *sodB*, *oxyR*, and *dps*, were upregulated. Hence, antibacterial activity of GO was associated with the GO-induced damage of cell membrane and disturbances in energy metabolism processes [179]. In addition, exposure to simulated sunlight considerably increased the antibacterial activity of GO. Since, under such conditions, only ^1^O_2_ showing minor impact on the oxidation of antioxidant biomolecules is generated, while oxidation is associated with light-induced electron–hole pairs, which are generated on the surface of GO, the light-induced electrons stimulate the reduction in GO, creating also additional carbon-centered free radicals contributing to improved antibacterial activities of GO. Therefore, GO-induced oxidative stress does not depend primarily on ROS, and light-promoted electron transfer from antioxidant biomolecules to GO causes damage to bacterial antioxidant systems and reduces GO [180]. Excellent antibacterial activity against *Xanthomonas oryzae* pv. *oryzae* exhibited GO applied at a dose of 250 μg/mL, which was able to kill 94.48% of cells, compared to bactericide bismerthiazol, achieving only 13.3% mortality [181]. Antibacterial activity of GO against Cu-resistant *Ralstonia solanacearum* was reported by Wang et al. [182].

Treatment of *P. syringae* and *Xanthomonas* *campestris* pv. *undulosa* bacterial pathogens as well as *Fusarium graminearum* and *F. oxysporum* fungal pathogens with 500 μg/mL GO killed approximately 90% of the bacteria and suppressed macroconidia germination by 80%, causing also partial cell swelling and lysis. Aggregated GO sheets were supposed to intertwine the bacterial and fungal spores by mechanically wrapping, causing disturbance of cell membranes; this resulted in reduced bacterial membrane potential and electrolyte leaching from fungal spores, resulting, ultimately, in cell lysis [183]. At exposure of *Aspergillus niger* and *Aspergillus flavus* to GO, a 62% reduction in biomass and abnormal hyphae was observed, causing apoptotic-like cell death, due to GO-induced oxidative stress. Moreover, lower levels of acid phosphatase, naphthol-ASBI phosphohydrolase, β-glucosidase, and β-galactosidase, i.e., enzymes involved in the catabolism of nutrients, were observed and production of volatile organic compounds by the fungi was affected as well. *A. flavus* was found to be more tolerant to GO than *A. niger* [184]. Besides, the powerful antifungal activity of nanoscale GO against *A. flavus* and *A. parasiticus*, GO can also be used as an adsorbent of aflatoxins; using a dose of 150 μg/mL GO, an effective reduction in aflatoxins was observed [185]. GO inhibited *Sclerotinia sclerotiorum* in Potato Dextrose Agar medium and exposure of *Brassica napus* seeds for 8–24 h and plants for 8–16 h to 15 mg/L GO resulted in inhibition of *S. sclerotiorum* growth, compared to the control, without damaging rapeseed plants [186]. Treatment with GO (62.5–500 μg/mL) remarkably reduced the mycelial biomass and branching of *F. graminearum* strain PH-1; affected expression of genes involved in the mycelial growth, cell wall development, and stress response; increased the histidine metabolism; reduced the number of lipid proteins involved in cell wall synthesis; increased the levels of glucose, succinate, citrate, γ-aminobutyric acid, glutamine, and trehalose; considerably reduced the lipids in the fungi; and remarkably reduced hypoxanthine and guanosine levels, which affected DNA and RNA synthesis [187]. According to Wang et al. [188], the antifungal activity of GO and rGO against *F. graminearum* and *Fusarium poae* was associated with their deposition on the surface of the spores, inhibiting water uptake and inducing plasmolysis. rGO inhibited the mycelial growth of *A. niger*, *Aspergillus oryzae* and *F. oxysporum* with IC_50_ values of 50, 100, and 100 μg/mL, respectively, and this inhibitory activity was associated with sharp edges of rGO, causing damage to the cell membrane [189]. Inhibition of *Botrytis cinerea* growth by rGO was reported by Hao et al. [190].

Treatment of *Lens culinaris* plants inoculated with *Meloidogyne incognita* and *Macrophomina phaseolina* with 500 ppm GO effectively reduced galling, nematode multiplication, and root-rot index (achieving value of 2). At application of lower doses of GO, greater reductions in galling, nematode multiplication, and root-rot index were observed; 250 ppm and 125 ppm GO reduced the root-rot index to 3 and 4, respectively [191].

GO−Ag nanocomposite exhibited four-fold higher activity against *Xanthomonas oryzae* pv. *oryzae*, compared to pure AgNPs; due to destruction of the cell integrity, leakage of intercellular contents, enhanced ROS, inhibition of DNA replication, and complete inactivation of bacteria was observed already at a dose of 2.5 μg/mL [192]. A nanocomposite consisting of DNA-directed AgNPs on GO significantly reduced *Xanthomonas perforans* cell viability in the culture and on plants, and already at a dose of 16 ppm, showed excellent antibacterial activity; in a greenhouse experiment, already a dose of 100 ppm pronouncedly suppressed the severity of bacterial spot disease, compared to untreated plants [193]. Ag−dsDNA−GO nanocomposite, designed as an alternative to Cu for treatment of bacterial spot in tomato plants caused by *Xanthomonas* spp., exhibited antibacterial activity against Cu-tolerant and-Cu-sensitive *X. perforans* as well as Cu-tolerant *X. vesicatoria*, *X. euvesicatoria*, and *X. gardneri* strains. In a greenhouse experiment, treatment of *Solanum lycopersicum* plants prior to artificial inoculation with Ag-dsDNA-GO, using a dose of 75 or 100 μg/mL, pronouncedly reduced disease severity compared Cu−mancozeb application [194]. CuO NPs loaded onto the surfaces of GO sheets exhibited 16-times higher antibacterial activity against *P. syringae* pv. *tomato* than Kocide^®^ 3000, and in an in vivo test were able to reduce the severity of bacterial speck below 25%, without exhibiting a phytotoxic impact on tomato plants already at doses of 4 μg/mL and 8 μg/mL, while a similar effect was achieved with 125 mg/mL and 250 mg/mL Kocide^®^ 3000 [195].

GO−AgNPs nanocomposite showed three- and seven-folds higher inhibition of *F. graminearum* in vitro and in vivo with minimum inhibitory concentration (MIC) related to spore germination inhibition of 4.68 μg/mL; the spores and hyphae were damaged via physical injury and ROS generation. Moreover, powerful antifungal activity of the composite was likely also associated with the reduction in GO by fungal spores. GO−AgNPs nanocomposite also effectively controlled the leaf spot disease in *F. graminearum*-infected detached leaves [196]. Effective suppression of germination of sporangia of *Plasmopara viticola* and grapevine downy mildew disease can be achieved with application of GO−Fe_3_O_4_ nanocomposite. Treatment of grapevine leaves with 250 μg/mL GO−Fe_3_O_4_ considerably reduced the severity of downy mildew, indicating curative impact of this nanocomposite, whereby not even a dose of 1000 μg/mL exhibited toxic effects on plants [197]. CuO NPs decorated with rGO, applied at a dose of 1 mg/L, exhibited higher antifungal activity against *F. oxysporum* in vitro than 2.5-fold higher dose of the conventional fungicide Kocide^®^ 2000, whereby CuO NPs−rGO caused death of fungal cells by creating pits and pores on the fungal cell membranes. In an experiment with *F. oxysporum*-infected tomato and pepper plants, a dose of 1 mg/L CuO NPs−rGO was able to reduce Fusarium wilt and root rot diseases severity by >5% for 70 days, whereby phytotoxic effects were not observed; with application of Kocide^®^ 2000 at a dose 2.5 mg/L, only 30% disease reduction was observed [198]. Combination of rGO with cationic polymer can improve the binding of rGO on leaf surface of chili crop by more than 30%, compared to control, and diminish its leaching in soil by 45% more than control. By decorating rGO with Cu_2_-xSe nanocrystals and subsequent coating of nanocomposite with CS, effective antifungal pH-responsive formulation was prepared, which was able to reduce the *Colletotrichum capsici* growth by similar to 1/2 times, compared to captan control [199]. Improved bamboo timber mold resistance can be achieved by coating it with rGO and nanocrystal ZnO nanocomposite, whereby *A. niger* mold resistance of nanocomposite was grade 2, compared to the grade 4 of original bamboo timber, and *Trichoderma viride* and *Penicillium citrinum* mold resistance of nanocomposite was even grade 0 [200].

GO combined with mancozeb, cyproconazol, and difenoconazole fungicides exhibited synergistic inhibitory effects on the mycelial growth and biomass as well as spore germination of *F. graminearum* compared to single fungicides, and also in a field experiment pronouncedly reduced Fusarium head blight disease incidence and disease severity in wheat plants. Upon GO-fungicide treatment, mycelia were shrunk and deformed, and membrane fusion, due to the changes in cell membrane permeability and loss of cell wall integrity, along with disappearance of cytoplasm, was observed [201]. The IC_50_ value related to the inhibition of mycelium growth of *Magnaporthe oryzae* by carbendazim−GO nanocomposite was 0.28 μg/mL, compared to 0.64 μg/mL estimated at single carbendazim application, suggesting 2.29-fold higher antifungal activity. GO can disrupt mycelia and threaten cell integrity, and can impair glutathione (GSH) on the cell membrane via electron transfer what results in reduced activity of fungal cells. Moreover, in greenhouse experiments this nanocomposite considerably reduced the severity of rice blast [202]. GO−polydopamine nanocomposite loaded with hymexazol showed NIR-laser-dependent and pH-dependent release of this fungicide as well as higher adhesion performance and persistence than the solution of hymexazol fungicide after a simulated-rainwash experiment, exhibiting antifungal activity against *F. oxysporum f.* sp. *cucumebrium,* which was comparable with that of hymexazol solution [203].

## 5. Effects of GBNs on Plants

As mentioned above, GBNs are intensively studied for their applications against a variety of crop pests. Just as various modified GBNs have positive and negative effects on healthy human cells, in addition to the desired antiproliferative/killing effects of bacterial or cancer cells, GBNs have both positive and negative effects on plant cells, affecting the performance of the whole plant. The latest findings on the effects of GBN on plants divided into negative and positive impacts are given below, and selected effects are summarized in Table 3.

### 5.1. Phytotoxic Impact

Mechanistic analysis of ecological effects of graphene nanomaterials on plant ecosystems, focusing predominantly on their harmful impact on physiology, biochemistry, and gene expression in terrestrial plants and supposed toxicity mechanisms, was presented by Yang et al. [226]. Application of 5 g/kg GR pronouncedly stimulated growth of *Medicago sativa* plants, while higher doses (10–20 g/kg) exhibited adverse impact and impaired physiological and morphologic characteristics of plants. A dose of 5 g/kg GR also considerably increased the tolerance of plants exposed to salt and alkali stresses, which was manifested with enhanced biomass and improved antioxidant-enzyme activities in plants [227]. Moreover, foliar application of GR improved photosynthesis and the antioxidative defense system as well as mitigated salinity and alkalinity stresses in *Medicago sativa* L. plants via regulating gene expression, suggesting adaptation of plants to these abiotic stresses [228]. The number of differentially expressed genes (DEGs) in *M. sativa* plants exposed to GR (10 or 20 g/kg) increased with increasing degree of abiotic stress; the GR-responsive genes were predominantly linked to the biosynthesis of amino acids, isoflavonoid biosynthesis, linoleic acid metabolism, and phenylpropanoid biosynthesis pathways as well as many other DEGs, such as nitrogen metabolism, photosynthetic, antioxidant enzyme, and metabolic sucrose and starch genes that can be involved in the response to GR. However, it was found that in the accommodation of *M. sativa* plants to GR stress, enzymes involved in nitrogen metabolism are decisive [229]. The growth of *Fagopyrum esculentum* plants exposed to GO (diameter: 0.5–3 µm, thickness: 0.55–1.2 nm; 10–500 mg/L) was inhibited in a dose-response manner and GO was assessed in roots and stems. At high GO concentrations (≥100 mg/L), enhanced ROS generation and regulation of activities and gene expression of oxidative enzymes was observed, suggesting that plant growth inhibition was due to regulation of ROS detoxification. The enrichment of GO-responsive genes in cell cycle and epigenetic regulation was higher in stem compared to root. Within 2039 GO-responsive DEGs 36 genes were involved in ROS detoxification. Moreover, considerable regulation of 40 genes involved in biosynthesis and the signaling of plant hormones, 97 small secreted peptides encoding genes as well as the gene expression of 111 transcription factors and 43 receptor-like protein kinases by GO were observed as well [230].

Surface functionalization of GRQDs affected their phytotoxicity to hydroponically cultivated *Lactuca sativa* plants. Carboxylated and hydroxylated GQDs applied at a dose 50 mg/L reduced dry root biomass by 39% and 43%, respectively, and shoot dry biomass by 44% and 36–55%, respectively, causing increased oxidative stress manifested in impairment of photosynthesis, modulating levels of phytohormones and disrupting homeostasis of nutrients, while aminated GQD increased root dry biomass by 34% and did not affect shoot dry biomass [207]. GR (25–500 mg/L) exhibited hormetic effect on dry biomass of roots, stems, and leaves of *Larix olgensis* plants, and inhibitory effects were observed at ≥100 mg/L GR. After one month of incubation, there was a reduction in photosynthetic pigments, soluble protein, and proline, while an increase in MDA and ROS levels was observed. However, presence of GR affected also soil properties via enhancing organic matter, hydrolytic nitrogen, and availability of P and K, and reducing activities of acid phosphatase, urease, dehydrogenase, and CAT [231]. GRQDs applied to *S. lycopersicum* (250–500 mgL) and *Vigna radiata* (250–1250 mg/L) plants cultivated in hydroponium, increased chlorophyll (Chl) levels in plants, while higher GRQDs doses (1000–1500 mg/L) inhibited growth and enhanced CAT and glutathione reductase activities as well as H_2_O_2_, MDA, and proline, plus GSH levels in both plants, with tomato seedlings being less sensitive to GRQDs-induced stress than mung bean plants [225]. Moreover, at exposure of *Betula pubescens* microclones to GO in the concentration range 1.5–15 μg/L, a hormetic effect was observed. GO was able to protect plants against phytopathogens at the stage of culture establishment and stimulated the shoot’s survival rate. Morphological characteristics of plants were stimulated at doses of 1.5 μg/L and 3 μg/L GO, but were inhibited by 15 μg/L GO. Survival rate of plantlets by GO treatment was also enhanced at the multiplication stage. GO improved photosynthetic and CAT activity of treated plants, reduced the number of stomata, and at the rooting stage a dose of 1.5 μg/L GO increased the number of plantlets with roots compared to control plants [215].

Whereas humic acid alleviated phytotoxic impact of GO, the nanocolloids adsorbed on GO entered algal cells similarly as pristine GO but generated higher ROS amounts, causing more serious DNA damage and plasmolysis as well as greater inhibition of photosynthesis than GO. Inhibition of carbohydrate, fatty-acid, and amino-acid metabolism was associated with higher ROS production [232]. Moreover, at exposure of cyanobacterium *Microcystis aeruginosa* to GO, the photosynthetic pigment content decreased (IC_50_ related to Chl*a*: 11.1 μg/mL). At treatment with 11.1 μg/mL GO, the esterase enzyme activity was reduced but remarkable membrane damage was not observed, suggesting absence of oxidative stress. Therefore, it can be supposed that in indirect toxicity of GO to *M. aeruginosa*, light shading and cell aggregation play a role [233].

*Plantago major* leaf-derived calli cultures grown on a 1/2 MS medium, which were exposed to drought stress and treated with 800 μg/mL GO, were characterized with reduced relative growth rate (78.5%), osmotic potential (48.2%), and increases in dry biomass matter (35.1%) and H_2_O_2_ (54.2%) levels, compared with control. A dose of 800 μg/mL GO increased also total phenolic content (40.9%) and total flavonoid content (35.3%) as well as remarkably diminished proline content (26.9%) at water deficit as well as under normal water availability conditions, when compared with respective controls [217]. Treatment of *Brassica napus* plants with 25 mg/L GO inhibited root development and generated oxidative stress, whereby its inhibitory effect increased with co-application of indole-3-acetic acid (IAA) (>0.5 mg/L). At such co-treatment of plants, the root growth of rapeseed was regulated via multiple phytohormone pathways, including those involving abscisic acid (ABA), IAA, gibberellin, cytokinin, brassinolide, and salicylic acid, and, especially, gibberellin content was affected [234].

Whereas Ag−GO nanocomposite stimulated growth of radish root at treatment with 0.2 mg/mL, and shoot growth stimulation was observed at the application of 0.2–1.6 mg/mL GO, alfalfa plant responded to treatment with 0.2–1.6 mg/mL with root growth inhibition. At exposure to Ag−GO, H_2_O_2_ was produced, and its increased accumulation in tested plants suggested the risk for the transfer Ag−GO to higher trophic levels [210]. Microwave-synthesized Ag−rGO nanocomposites (1–6 mg/L) incubated 24 h with *Chlorella vulgaris* exhibited dose-dependent toxicity against algae, which was manifested in morphological shrinkages of algal cells and alteration in the position of nucleoli; impaired growth characteristics; reduced contents of phenols, flavonoids, and photosynthetic pigments; and increased levels of H_2_O_2_ [235]. Exposure of bare-rooted seedlings of *Pinus tabuliformis* Carr. cultivated in pots for 6 months to 25 mg/L GO via irrigation resulted in growth stimulation, however, plasma-wall separation and destruction of membrane integrity in root cells was observed; based on differentially expressed genes, it was stated that plant responses to GO treatment resembled those observed under salinity stress, while responses to biotic stimuli were suppressed; changes in metabolic processes and hormone signal transduction pathways were estimated as well. Growth stimulating impact of GO was associated with increased and upregulated expression of the auxin response gene *SAUR41* and *PYL* genes, which encode ABA receptors, and glycogen synthase kinase 3 homologs [236].

Exposure to GO (200–800 mg/L) pronouncedly reduced NO_3_^−^ concentrations in roots of *Triticum aestivum* plants via considerable inhibition of net NO_3_^−^ influx in wheat roots. GO was trapped in the root vacuoles and remarkably diminished not only the root length but also the number of lateral roots, causing root-tip whitening, creases, and impaired respiration, preventing the increase in root uptake area and enhancing oxidative stress. Moreover, damage of DNA and pronounced downregulation of the expression of nitrate transporters genes (*NRT1.3*, *NRT1.5*, *NRT2.1*, *NRT2.3*, and *NRT2.4*) was observed under GO exposure, resulting in the inhibition of NO_3_^−^ uptake rate by plants [208].

As a response to the injection of GO, rGO, and GOQDs into stems of pepper plants, Ca content was reduced by 21.7–48.3%, intercellular CO_2_ concentration by 12.0–35.2%, transpiration rate by 8.7–40.2%, and stomatal conductance by 16.9–50.5%, along with the decreased uptake of GR-based nanomaterials, due to downregulation of endocytosis and transmembrane transport proteins. On the other hand, the activation of defense system was reflected in upregulation of gibberellin and abscisic acid receptor PYL8. Higher levels of the oxidative stress in fruits were observed using rGO and GOQDs compared to GO, and in the phytotoxicity and defense mechanisms, downregulation of carbohydrate and upregulation of amino acid metabolism played the crucial role [204]. On the other hand, surface-oxygen content affecting the biological impacts was found to be crucial factor for the phytotoxic impact of GR nanomaterials. Whereas GO reduced morphological characteristics of shoots in rice plants, this was not observed with rGO. Oxidative stress in shoots at exposure to GO was due to enhanced Fe translocation, and its subsequent accumulation in the above-ground plant part, which was supported by GO-induced acidification of growing medium. Beside the antioxidant regulators, protection of plants against Fe toxicity was also ensured by downregulation of metabolites, which were associated with Fe transport. Plant root exudates contributed to the reduction in toxic GO to nontoxic rGO [209].

Under the environmentally relevant concentrations, treatment with GO (1 mg/L) pronouncedly enhanced phytotoxic impact of As^3+^ and As^5+^ (1 mg/L) on tomato and wheat plants. Up-regulation of the aquaporin- and phosphate-transporter-related genes expression by GO resulted in the increased accumulation of As^3+^ and As^5+^ in plants and greater reduction in the macro-and micronutrient content, compared to treatment with As alone. Moreover, GO increased the oxidation stress in plants exposed to As stress and caused severe damage in root plasma membranes, thereby endangering As detoxification pathways, including As complexation with GSH and efflux [205].

### 5.2. Beneficial Impact

It was found that 50 mg/L of GR increased the overall length and volume of the root as well as the number of root tips and forks of *Zea mays* L. seedlings compared to the control group. The nitrogen and potassium content in the rhizospheric soil was also increased. Maize plants responded to the presence of GR with increased regulation of transcription factors, signal transduction of plant hormones, nitrogen and potassium metabolism, and increased production of secondary metabolites in the roots [237]. The growth of plants was affected by sizes of GRQDs: while GRQDs of ca. 10 nm effectively stimulated plant growth, no effect was observed with immense GR particles, showing dimensions of micrometers [223]. Multilayer (10–12 layers) GR nanoplatelets (diameter: 2 µm; thickness: 8–12 nm) applied at a dose of 250 mg/L and 500 mg/L, increased fruit yield and number of fruits per plant, in tomato plants inoculated with *F. oxysporum*, along with increasing total Chl and ascorbic acid levels and reducing contents of phenols and H_2_O_2_ as well as glutathione peroxidase (GPX) activity in the leaves of *F. oxysporum*-inoculated plants [238]. Lu et al. [239] reported that high amounts of GR allocated in leaves of *Oryza sativa* plants were passively transported to the chloroplasts (44%) and thylakoids (29%), respectively, and promoted production of adenosine triphosphate (ATP) compared to control due to GO-mediated-facilitated-electron transport in thylakoids of photosystem (PS) II as well as protection of PS II against oxidative stress by GO, which functioned as an ROS scavenger. Stimulated photosynthetic activity of chloroplasts by GO was observed both in vitro and in vivo. Alkyl functionalized GR nanoribbons (GNRs; 2–15 μm × 40–250 nm) foliar applied on sugarcane leaves reduced the negative impact of chilling on the photochemical efficiency of PS II and photosynthetic gas exchange, and enhanced levels of Chls and carotenoids in leaves. Higher non-photochemical quenching of PS II was due to enhanced carotenoid levels in leaves [240]. Ag−GR (40 and 60 mM) increased contents of Chl, total phenol and total protein, levels of soluble sugars, flavonoids and phenol as well as the contents of valuable secondary metabolites stevioside and rebaudioside, in *Stevia rebaudiana* [221].

Beneficial impact of priming of tomato seeds with 10–100 mg/L GO on the contents of Chl, vitamin C, β-carotene, phenols, and flavonoids in *S. lycopersicum* plants was reported by Lopez-Vargas et al. [222]; treatment with GO also increased activities of phenylalanine ammonia lyase (PAL) and some antioxidant enzymes. Improved germination as well as root and shoot growth of *Gossypium hirsutum* and *Catharanthus roseus* plants was observed upon treatment with GO. Moreover, plants grown in soil supplemented with GO were characterized with early flower development and higher flower production, and GO also protected the plants from adverse impact of abiotic (saline and drought) stresses [241]. GO supplemented to the growth medium of *Arabidopsis thaliana* and injected into the stem of the *Citrullus lanatus* plant increased the length of roots, the area of leaves, the number of leaves, and the formation of flower buds; in *C. lanatus* plants, GO accelerated the fruit ripening process, which was manifested by increased perimeter and sugar content of the watermelon fruits [242]. Comparison of responses of GO-tolerant and GO-sensitive *Oryza sativa* plants showed that treatments with 5–150 mg/L GO increased seed germination and root growth as well as inhibited shoot growth of all genotypes, but the GO-tolerant genotype was able to mitigate adverse effects of ROS generated by GO via increasing activities of antioxidant enzymes SOD, CAT, and peroxidase (POD) [243]. Enhancement of fresh and dry root biomass of tomato plants as well as contents of non-enzymatic antioxidants (ascorbic acid, GSH, phenols, and flavonoids), photosynthetic pigments, and proteins, plus activities of ascorbate peroxidase, POD, GPX, CAT and PAL at treatment with GO (10–1000 mg/L) was reported by Gonzalez-Garcia et al. [216]. Application of GO at doses of 50 mg/L and 100 mg/L ameliorated the shoot/stem growth via enhancing the cortical cells number, cross-sectional area, diameter, and vascular-column area, and induced the expression of root development-related genes (*SlExt1* and *LeCTR1*), thereby stimulating development of the root system; it increased surface area of root tips and hairs of tomato plants, biomass accumulation, and root auxin content, resulting in enhanced number of fruits and accelerated fruit ripening, compared to the control plants [224].

Whereas 25 mg/L GO exhibited enhanced inhibition of *Chlorella pyrenoidosa* algae under eight-day sunlight irradiation because of higher oxidative stress and more serious membrane damage, in the presence of Cu^2+^ ions, the resulting toxic impact on algae was reduced, likely due to inhibition of the photo-transformation of GO by Cu^2+^ ions or due the adsorption/retention of Cu^2+^ ions and subsequent formation of less toxic Cu-based NPs, such as Cu_2_O and Cu_2_S, on the photo-transformed GO [244].

Treatment of basil plants exposed to salinity stress (0 and 100 mM) with 50 mg/L of glycine betaine-functionalized GO mitigated the adverse impact of salinity on plants via improving morphological characteristics of plants, increasing the antioxidant enzymes activities as well as contents of photosynthetic pigments, phenols, proline, and some important constituents of the essential oil, along with decreasing MDA and H_2_O_2_ levels and improving of membrane stability index. On the other hand, treatment of plants with a double dose was phytotoxic [206]. *Pennisetum glaucum* plants foliar-treated twofold with 20 mg/L GO after 30 and 60 days of cultivation, in soils showing salinity of 3.16 d/Sm and 10.29 d/Sm, respectively, showed pronouncedly ameliorated plant growth, biomass accumulation and yield as well as reduced oxidative stress in plants, resulting in ameliorated tolerance and adaptability of pearl millet plants to salinity stress [245]. Proline-functionalized GO NPs applied at a dose 50 mg/L and 100 mg/L to *Dracocephalum moldavica* plants exposed to salt stress (50 and 100 mM) effectively mitigated adverse impact of high saline levels on plants, reduced oxidative stress in plants and improved morphological and physiological characteristics of stressed plants. In increasing activities of antioxidant enzymes and contents of secondary metabolites in essential oil as well as membrane stability in salt-stressed (100 mM) Moldavian balm plants, particularly a dose of 50 mg/L was favorable [212]. Positive impact of seed treatment with GO on morphological characteristics of tomato plants exposed to salt stress was also reported by Lopez-Vargas et al. [246].

Exposure of *Paeonia ostii* to GO under drought stress showed that GO inhibited evaporation of soil water and did not affect the soil pH but reduced ROS induction, relative electrical conductivity, and free proline content in plants, along with improvement of the antioxidant enzyme activities compared to control plants. In water-stressed plants exposed to GO improved photosynthesis, higher number of intact mesophyll cells and organelles and open stomata was observed, compared to plants missing GO treatment; changes in the expression patterns of genes required for lignin biosynthesis, photosynthesis-antenna proteins, carbon fixation in photosynthetic organisms, and glyoxylate and dicarboxylate metabolism were also detected. Due to electrostatic repulsion between GO and the roots, GO did not accumulate in of *P. ostii* plants [247]. Co-exposure of drought-stressed maize plants to GO and plant growth-promoting bacterium *Rhizobium* sp. E20-8 mitigated adverse impact of water deficit on plants, whereby GO ensured osmotic and antioxidant protection of plants, while *Rhizobium* sp. E20-8 alleviated negative impact of GO on biochemical processes of plants [248].

Foliar spraying of Cd-stressed *Lactuca sativa* plants with 30 mg/L GO improved photosynthetic processes in plants, decreased levels of O_2_^·^^−^ and MDA as well as activity of antioxidant enzymes, and increased plant biomass [213]. Lettuce plants cultivated in the presence of 2 mg/L Cd, which were sprayed with 30 mg/L GO, showed improved morphological characteristics of plants and reduced Cd accumulation in roots and leaves, along with an increase in soluble sugar, protein, and vitamin C content, thereby improving nutritional quality of plants. GO, which was taken up by leaves and transported to roots via phloem, diminished toxic impact of Cd on cell wall and membrane, chloroplasts, and starch granules. Reduced bioavailability of Cd was achieved via reduction in Cd^2+^ ions fixed by GO, which was absorbed by lettuce cells; moreover, GO by regulating metabolic pathways of plant can contribute to ameliorated Cd tolerance of lettuce plants [214]. Faster germination of rice seed germination as well as root growth at application of 10 mg/L GO was due to the improved water uptake, however, GO applied at doses 1 and 10 mg/L, respectively, was able to mitigate toxic impact of Cd (1 mg/L) on seed germination. However, increased membrane permeability at treatment with 10 mg/L GO resulted in increased Cd uptake by rice roots and shoots [249]. In Cd-stressed *Oryza sativa* plants treated with 400 mg/L GO, the transcript levels of Cd transporters (OsIRT1, OsIRT2, OsNramp1, OsNramp5, and OsHMA2) were reduced by 56–96%, along with 60% reduction in Cd levels compared to GO-untreated plants, although the plant growth was adversely affected [211]. Low GO concentrations enhanced the Cd tolerance of jute plants, stimulated plants growth and activities of antioxidant enzymes, along with reducing oxidative stress and increasing Cd uptake in plants, while a dose of 20 mg/L inhibited plant growth, reduced Cd uptake in plants, and enhanced oxidative stress [220]. Duckweed exposed to GO greatly enhanced Cd^2+^ influx, resulting in increased Cd accumulation in fronds and roots, and an addition of GO to Cd-stressed plants downregulated phagosome pathway as well as some key proteins, such as Stx7, Rab7, and Tubastatin B, whereby GO and Cd were attached on the cell surface of duckweed. GO was found to be suitable to be used in phytoremediation of Cd-polluted waters by duckweed [219]. It should be noted that important changes in the structure of GO were observed at exposure to UV radiation (28–74 μW/cm^2^; up to 120 h) manifested in eliminating −OH and C=O functional groups; such microstructural and compositional changes of GO can affect its stability and adsorption capacity at application in remediation of waters [250]. The acute toxic impact of Cd on *Scenedesmus obliquus* algae in the presence of GR-based nanomaterials was affected by oxygen-containing functional groups on their surface and decreased in the following order GO > GR > NH_2_-modified GO. The impact of such nanoscale materials on Cd toxicity depends on their capacity for adsorption of metal ions, their dispersibility in water as well as on the mode of their interaction with aquatic organisms [251].

Treatment of wheat plants with sulfonated GO (SGO; 50–250 mg/L) protected them against the toxic impact of nitrate (140 mM) and NH_4_^+^ (5 mM) via inducing expression levels of proteins in photosynthetic reaction centers, which reduced harmful radicals as well as enhanced SOD activity and ascorbate (AsA) regeneration, resulting in high rates of AsA/dehydroascorbate (DHA), reduced and oxidized glutathione ratio (GSH/GSSG), and GSH redox state. At application of 500 mg/L SGO, the excess of radicals produced by NH_4_^+^ was stopped, via the regeneration of AsA and POD activity, rather than GSH regeneration [252].

Porous carboxylated GO−CS spheres acted as adsorbents and were able to immobilize Cu^2+^ in soil and reduce its bioaccumulation in *Triticum aestivum* plants [253]. A dose of 300 μg/mL GO was found to function as an effective elicitor for in vitro production of phenolics and flavonoids and anthocyanins in callus culture of *Lepdium sativum* in vitro, and can also effectively alleviate the harmful impact of salt stress, mainly due to increased synthesis of phenolics and enhanced PAL activity [218]. Bi_2_O_3_/TiO_2_@rGO nanocomposite, showing peroxidase-like and SOD-like properties, which can bind to the bacterial outer membrane and produce huge amounts of ROS (O_2_^·^^−^, ·OH and H_2_O_2_), thereby changing the permeability of the cell membrane, effectively inactivated *P. syringae* pv. *tomato* DC3000 and down-regulated the virulence genes (*hrpS*, *corS*, *iaaL* and *flgG*), resulting in disturbance of the pathogenicity of the bacteria. Foliar spraying of tomato plants infected with this harmful bacterium with Bi_2_O_3_/TiO_2_@rGO nanocomposite (4%) not only mitigated adverse impact of pathogen but also exhibited beneficial impact on growth and photosynthetic processes as well as the content of thiols and activities of polyphenol oxidase (PPO), PAL, and POD as well as upregulated *PR-2* and *PR-13* genes, which contributed to enhanced disease resistance [254].

Pristine GO and GO, conjugated with CS and ethylenediaminetetraacetic acid, caused mortality of *M. incognita* 2nd juveniles. In an in vivo greenhouse experiment the most efficient reduction in numbers of 2nd juveniles, galls, females, egg masses, and the developmental stage nematodes showed GO−CS nanocomposite, which also pronouncedly enhanced CAT, POD, PPO, and SOD activities, suggesting enhanced plant systematic immune response as well [255].

## 6. Conclusions

Graphene is a relatively new material, which is characterized by unique properties, which predetermine its wide use, both in technical fields and in biomedical applications. It is very easy to functionalize and modify, and so a number of so-called graphene-based nanomaterials are prepared from graphene, which have also found widespread use in various economic areas, including industry, agriculture, and medicine. While the technical fields accept all these modifications and, immediately, try to use them for even better materials for various sensors, optoelectronics, or as battery components, there is still a big question over biological applications. Although graphene-based materials are also extensively investigated for various biological applications, such as their own biological effects, use as nanocarriers of bioactive agents, in photodynamic, photothermal therapy (i.e., as combination therapy or materials suitable for theranostic approach), or as scaffolds for tissue replacement in tissue engineering, the results are embarrassing and ambiguous. Definitely, graphene-based nanomaterials have an excellent ability to kill cells, regardless of whether they are plant, animal/human tissues, or microorganisms, and are characterized by a large area allowing good-drug or agrochemical loading. On the other hand, safe limits for the concentration of graphene and derived materials for any cells are not clearly established. Many results published so far come from in vitro studies and the results of these studies are very ambiguous. Likewise, the data related to ecotoxicity of these graphene-based nanomaterials in agriculture, their impacts on non-target organisms as well as their behavior/persistence in water and soil are frequently missing, and therefore a responsible approach at their introduction into practice is mostly desirable, to avoid potential risks for ecosystems. Based on known facts, it can be stated that the total resulting activity/action on cells/organisms depends on the actual functionalization and size of the particles and also on the method of production. For this reason, it seems to make sense to use graphene-based nanomaterials in combination with, e.g., nanoclays or metal nanoparticles, resulting in toxicity reduction or activity increasing, so that applied doses of graphene-based nanomaterials can be reduced. Thus, although GBNs generally represent a promising and growing group of nanomaterials with great potential for applications in the biomedical fields and agriculture (and there is a high presumption that they will be used), at present, more data need to be obtained from in vivo experiments in animal models before any valid conclusions can be drawn, regarding the real applications of these types of nanomaterials.

## Figures and Tables

**Table 1:** Effects of selected graphene-based nanomaterials on treatment of cancer, infectious diseases, and other diseases.

Nanocomposites	Effects	Refs.
GO	toxicity to MDA-MB-231 cells	[114]
GO	inhibition of *E. coli*	[121]
GO	inhibition of *E. coli* and *S. aureus*	[125]
GO	protection of A549 and TC28a2 cells against Rubella virus infection	[119]
GO	antiangiogenic effect in primary human endothelial Huvec cells	[128]
COOH-CO–^99m^Tc	imaging agent	[129]
Pluronic–COOH-GO–ZnO	toxicity to U87MG and U138MG cells	[130]
PEG–GO + NIR irradiation	growth inhibition of HT29 cells (wound closure ability)	[134]
GO, PCA–CA–GO, PCA–CA–FA–GO	inhibition cell cycle at G_2_/M phase of HepG2 cells depolarization of mitochondrial membrane potential upregulation of ROS	[137]
benzofurazans modified GO	inhibition of biofilm formation (*S. aureus*, *E. coli*, *P. aeruginosa*) cytotoxic to HCT-116 cells	[127]
functionalized (poly-l-lysine, CS, alginate, dextran, PEG, PVP, polyetherimide, AlO(OH) loaded with urease B, ovalbumin) GO	activation of cellular and humoral immunity	[122]
Ag−GO	kills *B. subtilis*, *E. faecalis*, MRSA, *S. aureus*, *E. coli*, *S. marcescens*, *Shigella* sp., *Salmonella* sp., *S. liquefaciens*, *Proteus* sp., *E. cloacae*, and *P. aeruginosa*	[140]
Cu−GO	inhibition of *S. mutans* biofilm formation alterations in biofilm architecture damaged production and distribution of exopolysaccharides dysregulated expression of exopolysaccharide-associated genes	[142]
Au–GO	antioxidative activity ↑ cell viability of MSCs ↓ activity of platelets ↑ cell motility and differentiation of various MSCs-derived cell types ↓ induction of fibrotic formation ↓ M1 macrophage polarization ↑ induction of M2 macrophage ↑ endothelialization	[145]
TiO_2_–GO + NIR irradiation	inhibition of *S. mutans* hyperthermia ROS generation	[143]
polyaniline–MnO_x_–GOQDs + irradiation (365 nm)	antimicrobial activity to *E.coli* and *S. aureus* oxidative stress via generation of ·OH radicals and photogenerated holes	[144]
PEG–GO loaded with anticancer drugs	controlled release of drugs and bioimaging	[132]
FA–GO loaded with Pt anticancer drugs	↑ cumulative release rate of drugsinhibition SKOV3 cells	[136]
FA–GO loaded with raloxifene	pH-dependent drug release cytotoxic to MCF-7 and MDA-MB-231 cells	[135]
mannose–CS–GO loaded with ulvan	pH-dependent-controlled release and targeted delivery to U87 cells	[131]
Pluronic−GO loaded with DOX	apoptosis of U251 cells impact on MAPK signaling pathway activation of caspase-3 in U251 cells	[139]
Au@Ag–Fe_3_O_4_–GO loaded with DOX	covalent binding to anti-HER2 antibody active and passive targeting of SKBR3 cells pH-dependent drug release	[146]
Fe_3_O_4_@PEG–GO loaded with DOX	pH-dependent drug release ↑ apoptotic effects against MCF-7 cells	[151]
PVP–Fe_3_O_4_–GO loaded with quercetin	pH-dependent drug release ↑ toxicity to MDA MB 231 cells	[147]
β-cyclodextrin–cholic acid–hyaluronic acid–Fe_3_O_4_–GO loaded with camptothecin	multiple targeting (hepatic, CD44-receptor) ↑ local chemo-photothermal effects apoptosis of hepatocellular carcinoma cells release of drug	[148]
SPION–PCL/CS–GO loaded with 5-FU + magnetic field	↑ tumor site temperature ↓ plating efficiency of the cells ↑ Bax/Bcl-2 ratio↓ growth of CT-26 cells	[149]
γ-Fe_2_O_3_–GO–MitP loaded with MTX	release of drug ↓ ATP production ↓ mitochondrial membrane potential impairment of mitochondrial functions activation of apoptosis	[150]
citric acid–CS–TEPA–rGO	inhibition of biofilm formation (*S. aureus*, *P. aeruginosa*) (wound dressings)	[155]
Ag–rGO	photoantimicrobial activities inactivation of *S. aureus* under blue-light irradiation	[160]
Zn–hydroxyapatite–rGO	↑ antibacterial activity ↑ enhanced alkaline phosphatase activity ↑ proliferation of mesenchymal stem	[161]
ZnO–rGO	enhanced catechin release at acidic pH antiproliferative effect inhibition of biofilm formation ↑ ROS generation ↑ cell membrane damage ↑ cytochrome C release ↑ apoptosis	[162]
FA–Au–rGO +NIR irradiation	improved generation of ^1^O_2_ high photothermal conversion efficiency destruction of MCF-7 cells	[159]
arabinoxylan–CS–rGO loaded with sulfadiazine	controlled release of drug	[156]
Pluronic–rGO loaded with buprenorphine	prolonged release of drug (treatment of chronic pain in osteoarthritis)	[157]
Pluronic–rGO loaded with lidocaine	prolonged release of drug (prolonged effects of local anesthesia)	[158]

**Table 2:** Effects of two-dimensional graphene-based nanomaterials on harmful insects.

GBNs	Insect	Dose of GBNs	Effects	Refs.
GRQDs−cysteine protease from seeds of *Albizia procera*	*Tribolium castaneum*, *Rhyzopertha dominica*	7 mg/g	↓ number of eggs and larvae ↑ larval mortality ↓ adult eclosion	[170]
GO	*Ostrinia furnacalis*	500, 1000, 2000 μg/g	↑ larval and pupal weights ↑ pupation rate ↓ larval development time ↑ cholesterol, lipids, and triacylglycerides ↑ trypsin-like serine protease, glutathione *S*-transferase, heat shock protein, and glycosyl-transferase	[164]
GO-cyhalothrin GO-bifenthrin GO-fenpropathrin	*Tetranychus urticae* Koch	37.5–300 μg/mL 31.25–250 μg/mL	↑ activity of pesticides (based on IC_50_ values) temperature-responsive release	[176]
GO	*Bombyx mori* ovary cell line larvae	>25 mg/L 25 mg/L	↑ oxidative stress, ROS, DNA damage ↓ gonadosomatic index ↑ oxidation levels and antioxidant enzyme activity ovary tissues ↓ number of oogonia and oocytes in ovarian tissues ↓ amount of spawning	[168]
GO	*Spodoptera frugiperda*	1000 μg/g of diet d.m.	↓ fecundity and fertility ↓ efficiency of food conversion into biomass ↓ maximal approximate digestibility	[169]
GO+β-cyfluthrin GO+monosultap GO+imidacloprid	*Ostrinia furnacalis*	12.5–100 μg/mL 62.5–500 μg/mL 125–1000 μg/mL	↑ mortality synergistic effect on insecticidal activity ↑ dehydratation of the insect shrinking of the cuticle and damage to cement layer structure	[177]

**Table 3:** Effects of two-dimensional graphene-based nanomaterials on plants.

GNMs	Plant (Cultivation Medium)	Dose of GNMs	Effects	Refs.
GO, rGO, GOQDs	*Capsicum annuum*; soil	0.25–25 mg/kg	**↓** intercellular CO_2_ **↓** Ca content **↓** transpiration rate **↓** stomatal conductance	[204]
GO	*Triticum aestivum*; hydroponics + 10 or 20 mg/L As^3+^ or As^5+^	10 mg/L	**↑** oxidative stress **↑** damage in root plasma membranes **↓** complexation of As with glutathione **↓** macro-and micro-nutrient content	[205]
glycine betaine−GO	*Ocimum basilicum*; hydroponics+ 50 and 100 mM NaCl	50, 100 mg/L	**↑** agronomic characteristics **↑** contents of Chl, phenols, and proline **↑** membrane stability **↑** activities of antioxidant enzymes **↓** MDA and H_2_O_2_ content	[206]
COOH−GQDsOH−GQDs	*Lactuca sativa*; hydroponics	50 mg/L	**↓** root and shoot d.w. **↓** photosynthesis **↓** mineral nutrition **↑** ROS modulation of levels of phytohormones	[207]
GO	*Triticum aestivum*; hydroponics	200–800 mg/L	**↓** net NO_3_^−^ influx in roots **↓** root length **↓** number of lateral roots **↓** root uptake area **↓** respiration **↑** DNA damage **↓** expression of nitrate transporters in roots	[208]
GO	*Oryza sativa*; hydroponics	100, 250 mg/L	**↓** shoot growth **↓** shoot biomass **↑** Fe translocation and accumulation **↑** acidification of medium downregulation of coumarins and flavonoids	[209]
Ag−GO	*Raphanus sativus* *Cucumis sativus* *Medicago sativa* *R. sativus, M. sativa, C. sativus*; seed treatment+cultivation on filter paper	0.2–1.6 g/L 0.2 g/L 0.8 g/L 0.2–1.6 mg/L 0.2–1.6 mg/L	**↑** shoot growth **↑** root growth **↓** root growth **↓** root growth **↑** H_2_O_2_ production	[210]
GO	*Oryza sativa* (Cd-stressed); soil	400 mg/L	**↓** transcript levels of Cd transporters **↓** net Cd influx of rice roots **↓** robustness of plants **↓** plant growth	[211]
proline-GO	*Dracocephalum moldavica*; hydroponics + 50 and 100 mM NaCl	50,100 mg/L	**↑** morphological parameters **↑** contents of Chl and proline **↑** Chl index (SPAD) **↑** membrane stability index **↑** antioxidant enzymes activities **↑** EO secondary metabolites **↓** MDA and H_2_O_2_ content	[212]
GO	*Lactuca sativa*; hydroponics with 2 mg/L Cd; foliar spraying	30 mg/L	**↑** total length, surface area, average diameter, and hair number of roots **↑** plant growth **↑** soluble sugar, protein, and vitamin C content **↓** Cd accumulation in plant organs **↑** plant tolerance to Cd	[213]
GO	Cd-stressed *Lactuca sativa*; hydroponics	30 mg/L	**↑** net photosynthetic rates **↑** stomatal conductance **↑** transpiration rates **↑** Chl content **↑** photochemical efficiency of PS II **↑** PET rates **↑** Rubisco levels **↑** plant biomass **↓** contents of O_2_^·−^, H_2_O_2_ and MDA **↓** activities of antioxidant enzymes	[214]
GO	*Betula pubescens*; Murashige and Skoog nutrient medium	1.5, 3 μg/L 15 μg/L 1.5–15 μg/L	**↑** shoot length **↑** number of leaves **↑** photosynthetic and CAT activity **↓** height of shoots **↓** number of stomata	[215]
GR	*Solanum lycopersicum*; foliar spraying	10–1000 mg/L	**↑** contents of phenols, flavonoids, ascorbic acid, and glutathione **↑** contents of photosynthetic pigments and proteins **↑** activities of APX, CAT, glutathione peroxidase and PAL	[216]
GO	*Plantago major*; callus, 1/2 MS medium + drought stress	800 g/L	**↓** growth rate and osmotic potential **↓** proline content **↑** dry matter and H_2_O_2_ **↑** TPC and TFC	[217]
GO	*Lepidium sativum*; callus	300 mg/L	**↑** TPC and TFC **↑** PAL activity **↑** DPPH activity **↑** callus fresh weight	[218]
GO	Cd-stressed *Lemna minor* (50 μM CdCl_2_); Datko liquid medium	100 mg/L	**↑** plant growth **↑** Cd content in fronds and roots up-regulation of 4471 genes down-regulation of 3230 genes	[219]
GO	Cd-stressed *Corchorus olitorius*; hydroponics	5 mg/L 20 mg/L	**↑** plant growth **↑** activities of antioxidant enzymes **↑** Cd uptake by plants **↑** Cd tolerance of plants **↓** oxidative stress **↓** plant growth **↓** Cd uptake **↑** oxidative stress	[220]
AgNPs-GR	*Stevia rebaudiana*; soil	40 mM 40, 60 mM	**↑** Chl content and increased **↑** contents of soluble sugars, flavonoids, phenols and proteins **↑** contents of stevioside and rebaudioside	[221]
GR	*Solanum lycopersicum* (priming)	10–100 mg/L	**↑** contents of Chl, vitamin C, β-carotene, phenols, flavonoids, and H_2_O_2_ **↑** activities of PAL), APX, glutathione peroxidase (GPX), SOD, and CAT	[222]
GR	*Zea mays*; soil	50 mg/L	**↑** root length, root volume, number of root tips, and forks upregulated expression of plant hormone signal transduction, nitrogen and potassium metabolism, and secondary metabolism in roots	[223]
GO	*Solanum lycopersicum;* Murashige and Skoog medium	50, 100 mg/L	**↑** shoot/stem growth **↑** biomass **↑** surface area of root tips and hairs **↑** expression of root development-related genes **↑** root auxin and number of fruits accelerated fruit ripening	[224]
GQDs	*Vigna radiata* *Solanum lycopersicum* *S. lycopersicum* *V. radiata*; hydroponics	250–1250 mg/L 250–500 mg/L 1000–1500 mg/L	**↑** Chl content **↑** Chl content **↑** contents of H_2_O_2_, MDA, proline, GSH **↑** glutathione reductase and CAT activities	[225]

## Data Availability

Not applicable.
